# Supplementary material for: From Cell Populations to Molecular Complexes: Multiplexed Multimodal Microscopy to Explore p53-53BP1 Molecular Interaction
Source: Int J Mol Sci. 2024 Apr 25;25(9):4672. doi: 10.3390/ijms25094672 (PMC11083188; doi:10.3390/ijms25094672)
Supplement: Supplementary file 1 [file ijms-25-04672-s001.zip › SupplementaryFigureCaptions.docx]

**Supplementary Figures**

**Figure S1.** **Representative image of irradiated MCF10A cells after 48h**. Shown are **(a)** γH2A.X, **(b)** p53, **(c)** PolII-S5p, **(d)** KI67, **(e)** DNA, and **(f)** 53BP1 signals. ROIs highlight micronuclei (white) and an aberrant metaphase (red). Scale Bar: 50 μm.

**Figure S2.** **Representative image of irradiated MCF10A cells after 24h**. Shown are **(a)** γH2A.X, **(b)** p53, **(c)** PolII-S5p, **(d)** KI67, **(e)** DNA, and **(f)** 53BP1 signals. Drawn regions **(g)** identify segmented cells with a late DDR phenotype (lower number and increased average size of 53BP1 foci). **(h)** Masks of the segmented γH2A.X foci. **(i)** Masks of the segmented 53bp1 foci. Scale Bar: 50 μm.

**Figure S3.** **Home-made holder for inverted microscope to clamp the 35mm sample dish between Exchange PAINT imaging steps**.

**Figure S4. Sequential imaging workflow of four targets by Exchange DNA-PAINT**. Four protein targets in cells were labelled with primary antibodies and their corresponding secondary antibody-conjugated with docking strand DNA sequences (D4, D3, D2, and D5). In the first imaging step D4 and D3 were acquired with ATTO655 and Cy3b imagers. Then, the ATTO655 imager strands were imaged sequentially by D2 and D5 strand type with wash steps between each imaging round. In the beads channel, FNDs spots positions were obtained from every DNA PAINT acquisition. FNDs were employed as reference markers for drift correction during single long acquisitions and between channels. The zoomed region of the highlighted area (white dashed box) showed channels alignment. All single-molecule images depicting each target were merged to obtain a multi-parameter super-resolved image. Scale bar 5 μm.
