# Supplementary figures and images for: From Cell Populations to Molecular Complexes: Multiplexed Multimodal Microscopy to Explore p53-53BP1 Molecular Interaction
Source: Int J Mol Sci. 2024 Apr 25;25(9):4672. doi: 10.3390/ijms25094672 (PMC11083188; doi:10.3390/ijms25094672)

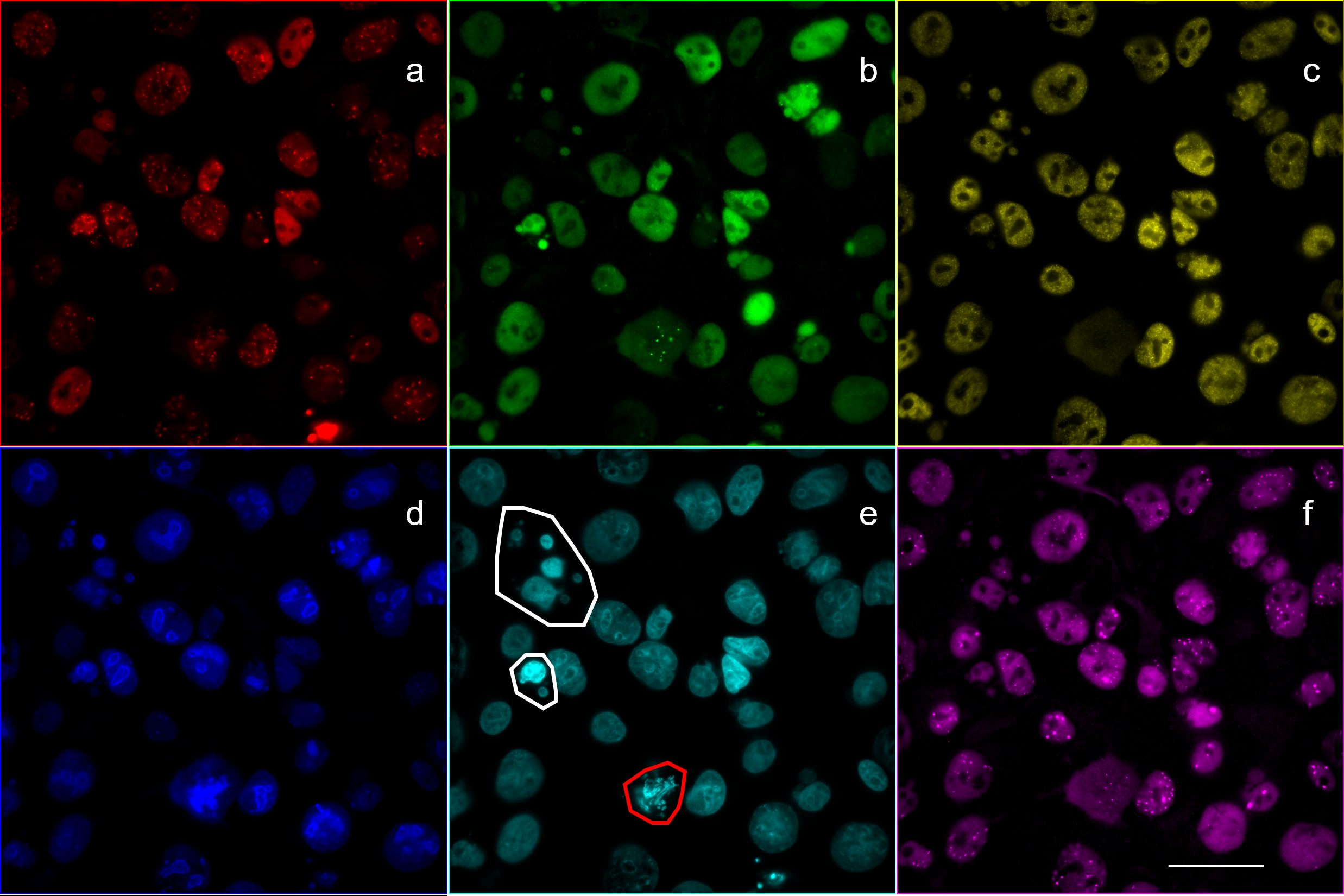

Supplement: Supplementary file 1 [file ijms-25-04672-s001.zip › Figure S1_revised.tif]

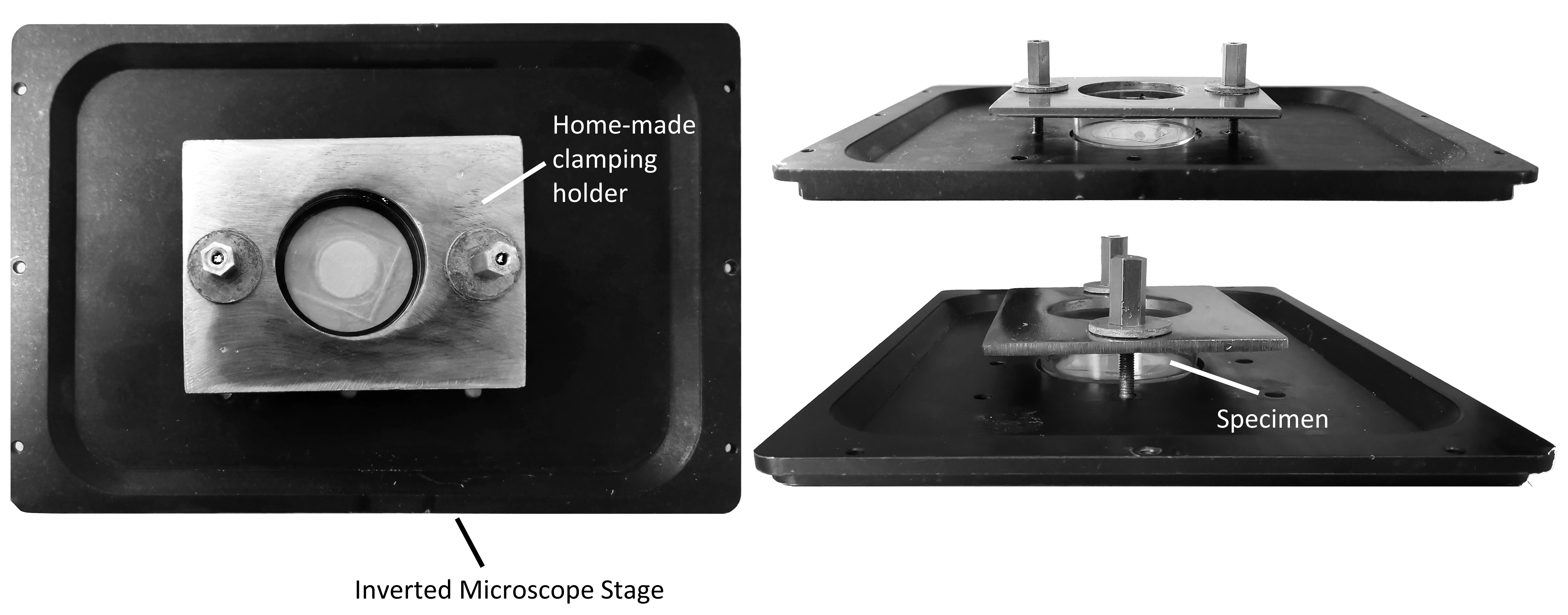

Supplement: Supplementary file 1 [file ijms-25-04672-s001.zip › Figure S3_revised.tif]

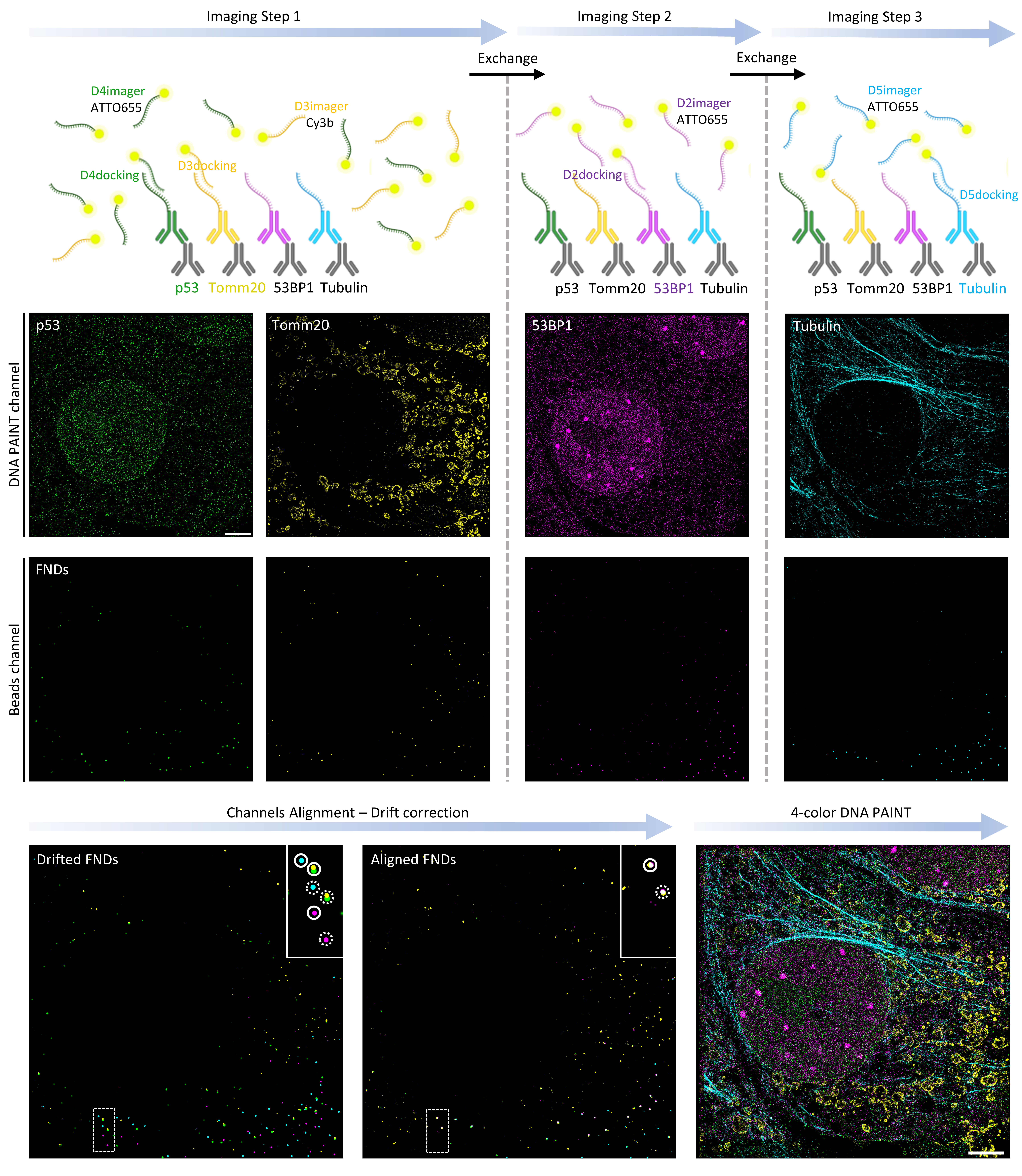

Supplement: Supplementary file 1 [file ijms-25-04672-s001.zip › Figure S4_revised.tif]
